# Pharmacological treatments and medication-related problems in nursing homes in Catalonia: a multidisciplinary approach

**DOI:** 10.3389/fphar.2024.1320490

**Published:** 2024-03-11

**Authors:** Emilie Anderssen-Nordahl, Margarita Sánchez-Arcilla Rosanas, Montserrat Bosch Ferrer, Mònica Sabaté Gallego, Eladio Fernández-Liz, Antonio San-José, Maria Estrella Barceló-Colomer

**Affiliations:** ^1^ Clinical Pharmacology Service, Vall d’Hebron University Hospital, Vall d’Hebron Barcelona Hospital Campus, Barcelona, Spain; ^2^ Clinical Pharmacology Group, Vall d’Hebron Research Institute, Barcelona, Spain; ^3^ Department of Pharmacology, Therapeutics and Toxicology, Universitat Autònoma de Barcelona, Barcelona, Spain; ^4^ Geriatric Unit, Internal Medicine Service, Vall d'Hebron University Hospital, Vall d’Hebron Barcelona Hospital Campus, Barcelona, Spain; ^5^ Primary Healthcare Barcelona, Management of Primary Care and the Community of Barcelona City, Catalan Institute of Health, Barcelona, Spain; ^6^ Foundation University Institute for Research in Primary Healthcare Jordi Gol i Gurina (IDIAPJGol), Barcelona, Spain

**Keywords:** medication review, frail elderly, nursing homes, medication therapy management, polypharmacy, potentially inappropriate medication list, primary healthcare, drug utilization

## Abstract

**Background:** Aging correlates with increased frailty, multi-morbidity, and chronic diseases. Furthermore, treating the aged often entails polypharmacy to achieve optimal disease management, augmenting medication-related problems (MRPs). Few guidelines and tools address the problem of polypharmacy and MRPs, mainly within the institutionalized elderly population. Routine pharmacological review is needed among institutionalized patients. This pharmacological review may improve with a multidisciplinary approach of a collaboration of multiple health professionals. This study aimed to describe institutionalized patients, systematically review their medication plans, and then give recommendations and identify MRPs.

**Methods:** A cross-sectional study was performed using data obtained from patients living in five nursing homes in the northern area of Barcelona, Spain. The inclusion criteria comprised institutionalized patients with public health coverage provided by the Health Department of Catalonia. A detailed description of the clinical characteristics, chronic diseases, pharmacological treatments, recommendations, incomplete data, and MRPs, such as potential drug–drug interactions, therapeutic duplications, contraindications, and drugs deemed inappropriate or of doubtful efficacy, was made. The clinical pharmacologist was the medical doctor specialist who acted as the coordinator of the multidisciplinary team and actively reviewed all the prescribed medications to make recommendations and detect MRPs.

**Results:** A total of 483 patients were included. Patients had a mean age of 86.3 (SD 8.8) years, and 72.0% were female individuals. All patients had at least three health-related problems, with a mean of 17.4 (SD 5.6). All patients, except one, had a minimum of one prescription, with a mean of 8.22 drugs prescribed (SD 3.5) per patient. Recommendations were made for 82.4% of the patients. Of these recommendations, verification of adequate use was made for 69.3% and withdrawal of a drug for 49.5%.

**Conclusion:** This study demonstrates a high prevalence of health-related problems and several prescribed drugs in nursing homes in Catalonia. Many recommendations were made, confirming the increased proportion of polypharmacy, MRPs, and the need for standardized interventions. A multidisciplinary team approach, including general practitioners, geriatric assessments, a clinical pharmacist, and a clinical pharmacologist, should address this problem.

## 1 Introduction

Advances in research and medical care have increased life expectancy, and the aging of the population is expected to increase significantly in the coming decades (Guisado-Clavero et al., 2019; Zito et al., 2023). In 2022, more than one-fifth (21.1%) of the European Union population was aged 65 or over, and the elderly are expected to account for 31.3% by 2100 (Eurostat, 2023). Longevity correlates with the incidence of chronic disease, and 55% to 98% of elderly adults suffer from multi-morbidity (Guisado-Clavero et al., 2019). Multi-morbid and frail patients likely require multiple medications to achieve optimal disease management (Herr et al., 2015; Hilmer and Gnjidic, 2017). Increased exposure to complex drug regimens involving ≥5 drugs, known as polypharmacy, or excessive polypharmacy, as in patients treated with 10 or more medications concomitantly, raises the risk of adverse events (Stuhec et al., 2021). Polypharmacy can also affect drug safety due to potentially inappropriate medications (PIMs), adverse drug reactions (ADRs), and the risk of interactions (Burato et al., 2021; Zhang et al., 2022; Doumat et al., 2023; Reinhild Haerig et al., 2023).

A medication-related problem (MRP) is an occurrence that involves drug therapy that can potentially interfere with health outcomes. Some MRPs are therapeutic duplications, potential drug–drug interactions (DDIs), potentially inappropriate medications (PIMs), and contraindicated drugs (Troncoso-Mariño et al., 2021).

Given the impact of inappropriate prescription in elderly patients, different tools have been proposed to help optimize the use of medications in older patients, such as the Beers criteria, STOPP/START, PRISCUS, Medication Appropriateness Index, Drug Burden Index, and anticholinergic risk scale, to assess the anticholinergic load, among others (Hilmer et al., 2007; Rudolph et al., 2008; Lunghi et al., 2022; By the 2023 American Geriatrics Society Beers Criteria^®^ Update Expert Panel, 2023; Mann et al., 2023; O’Mahony et al., 2023). According to the Catalan Health Service instruction 04/2012, all patients on chronic treatment should undergo a pharmacological review at least every year (Department of Health. Government of Catalonia, 2014).

Generally, the guidelines poorly consider the situation of the elderly with multi-morbidity (Guisado-Clavero et al., 2019; Zito et al., 2023). Furthermore, there is little information on patients in nursing homes with greater fragility and multi-morbidity, even though they present more polypharmacy, ADRs, and prevalence of interactions (Herr et al., 2015; Hilmer and Gnjidic, 2017). Some studies suggest deprescribing may be safe, feasible, well-tolerated, and beneficial for the elderly, and collaboration with clinical pharmacists can reduce polypharmacy and improve adherence to treatments (Ibrahim et al., 2021; Saeed et al., 2022). The transition of patient care between different healthcare settings can be a challenge due to elevated medication errors, but proper medication reconciliation during the transition could lead to fewer MRPs (Stuhec and Batinic, 2023).

A multidisciplinary approach, with an interprofessional collaboration, allows the sharing of clinical knowledge and different perspectives about institutionalized patients to improve their pharmacological treatments (Disalvo et al., 2020; Lunghi et al., 2022; Song et al., 2023). Data from patients with the highest multi-morbidity are essential for the provision of adequate healthcare to patients with multiple chronic conditions. This is in line with the findings of previous reviews highlighting the lack of intervention studies aimed at improving adequate polypharmacy in elderly patients (Saeed et al., 2022).

In addition, the care of institutionalized patients was a great challenge during the SARS-CoV-2 pandemic, with an increase in morbidity and mortality in nursing homes. Compared to previous years, the mortality in nursing homes was almost 10 times higher, and 71.9% of all deaths in Spain during COVID-19 were seen in nursing homes (Mas Romero et al., 2020; Ordovás et al., 2020; Rada, 2020; Arnedo-Pena et al., 2022). For this reason, a multidisciplinary team was created in Catalonia, Spain, to make a structured intervention in nursing homes. The intervention consisted of developing an improvement plan, reviewing the validity of prescriptions and medication plans, and detecting MRPs.

Therefore, the main objective of this study was to describe institutionalized patients and systematically review their medication plans in nursing homes in Catalonia. The secondary objectives were to describe the recommendations given and identify MRPs by analyzing whether the prescribed treatments can be considered adequate and safe, inappropriate, or have safer alternatives.

## 2 Methods

### 2.1 Study design and setting

The multidisciplinary intervention was a multicenter before–after study without a control group. As the first step of this intervention, a cross-sectional study was carried out to make this descriptive analysis. From a total of 48 nursing homes, the data were collected from 5 nursing homes, where the intervention was made, in the northern area of Barcelona, Spain. These 5 nursing homes were prioritized by the health administration during the intervention since it was considered that the patients in these nursing homes would benefit the most. The health administration selected these nursing homes because of their size, efficiency, and to cover the highest population percentage. With this selection, even though it was only 5 nursing homes, the intervention covered 22.3% of the residents in the nursing homes. The study population included all patients currently admitted to a nursing home at the start of this intervention, which was initiated on 1 July 2020 and ended on 1 February 2022. The inclusion criteria comprised institutionalized patients with the public health coverage provided by the Catalan Health Service. The exclusion criteria comprised institutionalized patients with health coverage provided by other insurers, a short-term life expectancy, hospitalization during the intervention, patients who died or were discharged in the first month of the review, and those who could not be intervened due to lack of information. There was no formal sample size calculation since the descriptive analysis was done on all the reviewed patients except those who were excluded.

The multidisciplinary team included general practitioners, nurses, social and administrative workers from primary care, clinicians and nurses assigned to the nursing homes, a clinical pharmacist, and a clinical pharmacologist. The pharmacist and clinical pharmacologist acted as consultors. However, it should be pointed out that the clinical pharmacologist was the medical doctor specialist who acted as the coordinator of the multidisciplinary team and actively reviewed all the prescribed medications to make recommendations. Hence, medication reconciliation was carried out by the clinical pharmacologist at the beginning of the medication review. Medication review is an essential part of medical practice, and it is contemplated within the activities of medical professionals to ensure the rational use of medication, considering the universal health coverage in Spain (Department of Health. Government of Catalonia, 2022). The main sources of information used by the clinical pharmacologist to conduct the review and give recommendations comprised the information contained in the technical data sheets, the support tools Self-Audit and PREFASEG (PREscripción FArmacéutica SEGura) (Pons-Mesquida et al., 2021; 2022), and the list of potentially inappropriate drugs proposed by the Catalan Health Service (Department of Health. Government of Catalonia, 2014; Catalan Health Service. Department of Health, 2020).

The support tools are Self-Audit and PREFASEG (PREscripción FArmacéutica SEGura, i.e., safe pharmaceutical prescription). Self-Audit identifies and resolves safety MRPs systematically. It generates a list of patients with active MRPs to facilitate changes or suspensions of a treatment (Pons-Mesquida et al., 2022). PREFASEG generates online notifications when starting a treatment to warn clinicians of potential problems related to drug use and prevent medication errors (Pons-Mesquida et al., 2021). The computerized medical history notifies the professionals when a patient is visited by another professional and explains the medication changes made.

The criteria used to consider MRPs were those established by the Catalan Health Service from recommendations on potentially inappropriate drugs in the elderly (Catalan Health Service. Department of Health, 2020) and the document on the management of medication in chronic patients (Department of Health. Government of Catalonia, 2014). These documents were prepared by consensus of a group of experts, and the criteria of the drugs to be included in the potentially inappropriate drug list were to be in at least two bibliographic databases, with an explicit recommendation or contraindication for the elderly population in the technical sheet or with a specific alert from the Spanish Agency for Medicines and Health Products (AEMPS, Agencia Española de Medicamentos y Productos Sanitarios). The references used were the Beers criteria, STOPP/START, the EU-PIM list, the PRISCUS list, information notes on medicines for human use from AEMPS, and anticholinergic risk scales in older adults (Department of Health. Government of Catalonia, 2014; Catalan Health Service. Department of Health, 2020).

From the identified problems during the medication review, different recommendations were given. These recommendations could be to complete absent data, withdraw a drug, verify whether the use of a drug was adequate, or substitute a drug. As for the missing data, allergies or diseases could be absent. As for the withdrawal of drugs, this was recommended when MRPs were considered, such as potential DDIs, duplicated therapies, contraindicated drugs, inappropriate drugs, or drugs of doubtful efficacy. As for the adequacy of drug use, this could be due to the need to reduce the dose, a bad tolerance, to reduce anticholinergic load, or a high risk of ADRs. As for the substitution of a drug, this could be recommended due to considering other drugs as a first choice or equivalent drugs.

The study design, procedures, and reporting followed the TREND guidelines for non-randomized evaluations of behavioral and public health interventions (Des Jarlais et al., 2004) and are registered at ENCePP (Reference: EUPAS106748).

### 2.2 Variables and data collection

The variables analyzed were demographic data; comorbidities; drug allergies; diseases according to the International Classification of Diseases, version 10 (ICD-10); pharmacological treatments according to the Anatomical Therapeutic Chemical (ATC) classification system; and the use of absorbents. The pharmacological treatments are recorded as the number of drugs consumed. This is the number of different drugs that the residents have prescribed, including fixed-dose combinations.

A descriptive analysis was performed of the recommendations, incomplete data, and drugs recommended to verify the adequacy of use, to be substituted, or withdrawn. We defined MRPs, potential DDIs, therapeutic duplications, contraindications, and drugs deemed inappropriate or of doubtful efficacy to identify deficits in functioning and analyze whether the prescribed treatments were considered adequate.

Comorbidities were collected according to the adjusted morbidity groups (AMGs) (Monterde et al., 2016) and complex chronic patients or a model of attention to advanced chronicity (Department of Health. Government of Catalonia, 2017).

AMG is a morbidity measurement created by the Spanish Healthcare System. This tool divides patients into 31 mutually exclusive categories from six morbidity groups (MGs) and five complexity levels (A) each (Monterde et al., 2016). This grouping aims to help identify patients with greater comorbidities, polypharmacy, risk of complications, worsening of functional capacity, quality of life, and/or premature death (Department of Health. Government of Catalonia, 2017).

The morbidity groups are as follows:

- MG = 0: Healthy population.
- MG = 10: Patients with an acute disease.
- MG = 20: Patients with a pathology related to pregnancy and/or birth.
- MG = 31: Patients with one system affected by a chronic disease.
- MG = 32: Patients with two or three systems affected by a chronic disease.
- MG = 33: Patients with four or more systems affected by a chronic disease.
- MG = 40: Patients with an active neoplasm.

The level of complexity takes into account the total of each morbidity group from the entire population used for its creation and divides it into five groups according to the percentiles 40, 70, 85, and 95 (Monterde et al., 2016). When AMG was compared to the clinical risk group measurement, the results showed better performance of AMG for Primary Healthcare in Spain (Hughes et al., 2004; Monterde et al., 2019).

A patient is considered to be a complex chronic patient when their clinical management is perceived as especially difficult by their referring clinical professionals. A complex chronic patient is associated with criteria related to the patient himself, clinical professionals, and the environment. Concerning the patient, there is multi-morbidity, severe or progressive single chronic pathology, a high probability of suffering decompensation, high use of health services, and polypharmacy, among others. Regarding clinical professionals, there is the requirement for multidisciplinary management, exposure to discrepancies between different professionals, management doubts, and benefits from an integrated care strategy. As for the social sphere, it is worth noting adverse psychosocial situations. No specific criteria or number are needed, rather than their referring professional considering the case management especially difficult.

A patient is considered to be in the model of attention to advanced chronicity when characterized by a case management approach with a present, important, and growing palliative pathway. The palliative component does not exclude curative options but rather coexists with them and advances decision planning as an essential process in decision-making support (Department of Health. Government of Catalonia, 2017).

The data were collected in the usual clinical practice during the intervention, and the data source was the electronic medical record that is common in Catalonia. Then, anonymized data were entered into the Research Electronic Data Capture (REDCap) platform. A quality check was done prior to the descriptive analysis. A detailed description of the clinical characteristics, chronic diseases, and pharmacological treatments was made.

### 2.3 Ethics approval

The study was conducted according to the guidelines of the Declaration of Helsinki. The protocol was approved by both local Research Ethics Committees of Vall Hebron University Hospital (protocol code EOM(AG)067/2021(5930)) and IDIAP Jordi Gol (protocol code 22/027-P). No informed consent was necessary since the information was anonymized.

### 2.4 Statistical analysis

Continuous variables are presented as means (standard deviation, SD), and categorical variables are presented as frequencies (percentages). Statistical analysis was performed using R version 4.3.0.

## 3 Results

### 3.1 Descriptive analysis of the institutionalized patients

**FIGURE 1:**
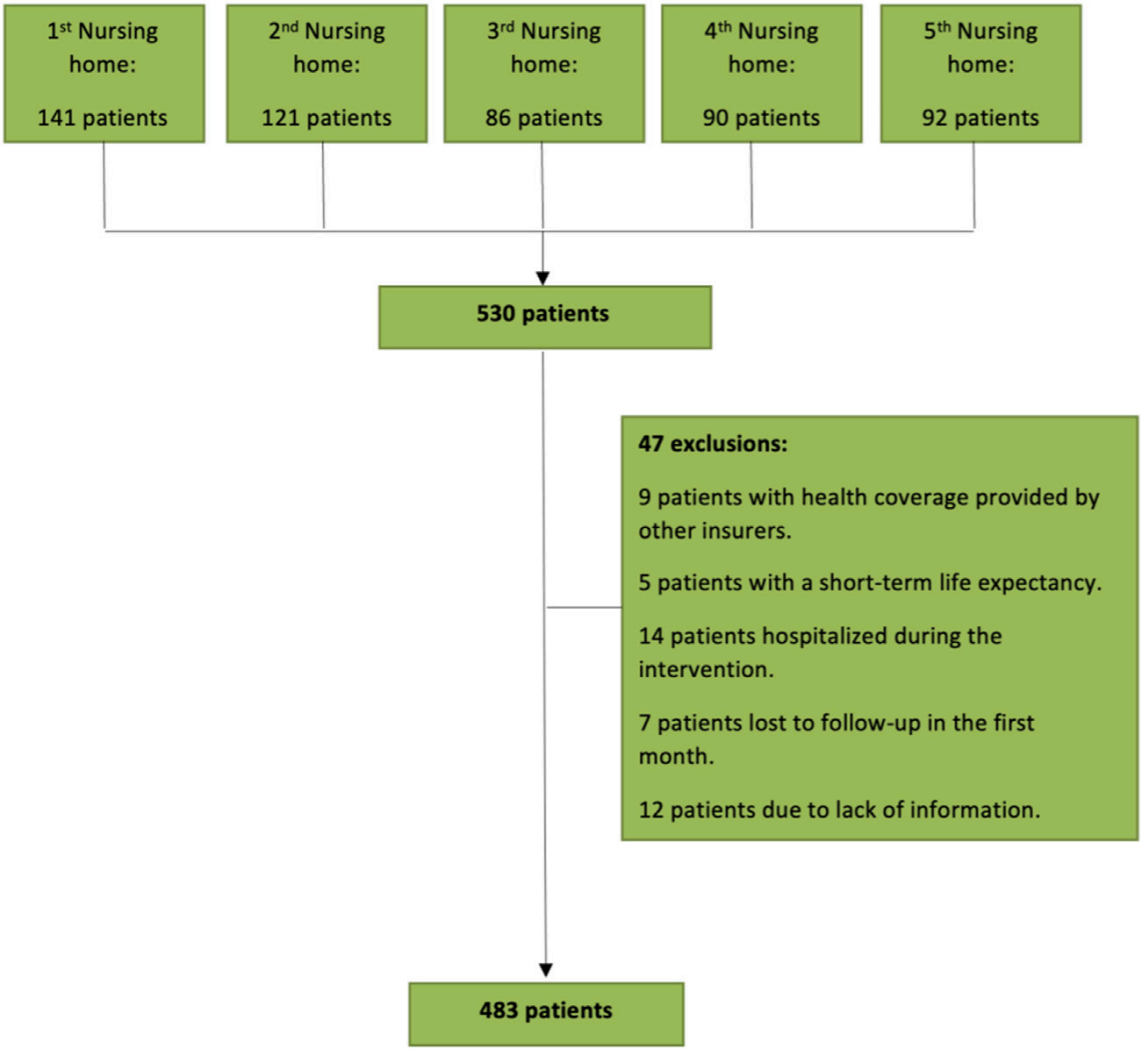
Flowchart of the study population the inclusion/exclusion procedure.

A total of 483 patients were included from five different nursing homes after excluding 47 patients (Figure 1).

The baseline characteristics of all the included patients are shown in Table 1. The patients had a mean age of 86.3 (SD 8.8) years, and 348 (72.0%) patients were female individuals. Complex chronic patients or patients of the model of attention to advanced chronicity were recorded in less than 2.0%, and almost 95.0% of the patients were in the morbidity group of patients, with four or more systems affected by chronic disease (MG = 33), in all nursing homes.

**TABLE 1:** Baseline clinical characteristics of the included patients.

Baseline clinical characteristic	Total	Residency 1	Residency 2	Residency 3	Residency 4	Residency 5
Number of patients	483	129 (26.7%)	111 (22.9%)	74 (15.3%)	81 (16.7%)	88 (18.2%)
Age (years)	86.3 (8.8)	86.2 (9.8)	87.9 (8.1)	84.6 (10.2)	87.2 (7.4)	84.8 (7.6)
Sex						
*Female*	348 (72.0%)	100 (77.5%)	86 (77.5%)	47 (63.5%)	56 (69.1%)	59 (67.0%)
*Male*	135 (28.0%)	29 (22.5%)	25 (22.5%)	27 (36.5%)	25 (30.9%)	29 (33.0%)
Complex chronic patients or advanced chronicity						
*Yes*	6 (1.2%)	0 (0.0%)	1 (0.9%)	1 (1.4%)	1 (1.2%)	3 (3.4%)
*No*	2 (0.4%)	0 (0.0%)	1 (0.9%)	0 (0.0%)	1 (1.2%)	0 (0.0%)
*Not recorded*	475 (98.3%)	129 (100.0%)	109 (98.2%)	73 (98.6%)	79 (97.5%)	85 (96.6%)
Recorded AMGs						
*Yes*	380 (78.7%)	111 (86.0%)	98 (88.3%)	42 (56.8%)	55 (67.9%)	74 (84.1%)
*Exitus*	86 (17.8%)	14 (10.9%)	12 (10.8%)	26 (35.1%)	24 (29.6%)	10 (11.4%)
*Not recorded*	17 (3.5%)	4 (3.1%)	1 (0.9%)	6 (8.1%)	2 (2.5%)	4 (4.5%)
*Risk of hospitalization in %*	11.5 (5.9)	12.8 (6.1)	9.7 (4.3)	9 (5.3)	11.8 (5.9)	13.1 (6.7)
Value of MG						
*MG = 40*	11 (2.9%)	2 (1.8%)	2 (2.0%)	1 (2.4%)	4 (7.2%)	2 (2.7%)
*MG = 33*	359 (94.5%)	106 (95.5%)	91 (92.9%)	40 (95.2%)	50 (91.0%)	72 (97.3%)
*MG = 32*	9 (2.3%)	2 (1.8%)	5 (5.1%)	1 (2.4%)	1 (1.8%)	0 (0.0%)
*MG = 31*	1 (0.3%)	1 (0.9%)	0 (0.0%)	0 (0.0%)	0 (0.0%)	0 (0.0%)
Drug allergies						
*Yes*	36 (7.5%)	32 (24.8%)	1 (0.9%)	2 (2.7%)	1 (1.2%)	0 (0.0%)
*No*	324 (67.1%)	52 (40.3%)	65 (58.6%)	70 (94.6%)	51 (63.0%)	86 (97.7%)
*Not recorded*	123 (25.5%)	45 (34.9%)	45 (40.5%)	2 (2.7%)	29 (35.8%)	2 (2.3%)
Number of health problems	17.4 (5.6)	17.9 (5.5)	16.6 (5.3)	15.7 (5.0)	16.2 (4.6)	20.4 (6.4)
Use of absorbents						
*Yes*	374 (77.4%)	98 (76.0%)	75 (67.6%)	52 (70.3%)	69 (85.2%)	80 (90.9%)
*No*	109 (22.6%)	31 (24.0%)	36 (32.4%)	22 (29.7%)	12 (14.8%)	8 (9.1%)
Number of drug consumption	8.22 (3.5)	8.1 (3.1)	7.7 (3.4)	8.6 (3.9)	8.2 (3.1)	8.8 (3.8)

*Numeric variables: mean (SD) and categorical variables: n (%).

All patients had at least three health-related problems (HRPs), with a mean of 17.4 (SD 5.6). The most common chronic diseases were urinary incontinence, with a total of 412 patients (85.3%), followed by hypertension, with 357 patients (73.9%), and osteoarthritis, with 264 patients (54.7%), as seen in Table 2. There was a total of 8419 HRPs documented, showing that a patient normally had various HRPs registered in the superfamilies. The number and percentage of the total registered diseases divided into superfamilies are shown in Table 3. For a complete list of all HRPs divided into groups according to their ICD-10, see Supplementary Table S1. In 197 (40.8%) patients, COVID-19 was registered as an HRP.

**TABLE 2:** Summary of the 40 most frequent chronic diseases and health-related problems.

Diseases and health-related problems	n	%
Urinary incontinence	412	85.3%
Hypertension	357	73.9%
Osteoarthritis and other arthritis	264	54.7%
Dyslipidemia	260	53.8%
Alzheimer’s disease or dementia	255	52.8%
Anemia	252	52.2%
Insomnia and sleep disorders	181	37.5%
Problems related to care provider dependency or life-management	166	34.4%
Functional intestinal disorders	146	30.2%
Symptoms and signs involving cognitive functions and awareness	146	30.2%
Diabetes mellitus	144	29.8%
Depression	138	28.6%
Atrial fibrillation and flutter	135	28.0%
Chronic kidney disease	134	27.7%
Injury of a body region	133	27.5%
History of any surgical intervention	131	27.1%
Osteoporosis	130	26.9%
Pressure ulcer	122	25.3%
Varicose veins or other disorders of veins	122	25.3%
Skin changes or soft tissue disorders	120	24.8%
Heart failure	119	24.6%
Malignant neoplasm	117	24.2%
Pain	108	22.4%
Dependence on enabling machines and devices	104	21.5%
Age-related cataract	100	20.7%
Altered laboratory findings	100	20.7%
Vitamin D deficiency	99	20.5%
Cerebral infarction	97	20.1%
Personal history of allergy to drugs	93	19.3%
Hearing loss	90	18.6%
Dermatitis and eczema	89	18.4%
Abnormalities of gait and mobility	88	18.2%
Glaucoma	87	18.0%
Chronic obstructive pulmonary disease	87	18.0%
Hernia	85	17.6%
Fecal incontinence	85	17.6%
Overweight and obesity	83	17.2%
Fracture of femur or pelvis	80	16.6%
Hypothyroidism	73	15.1%
Infections	70	14.5%

**TABLE 3:** List of all the registered health-related problems divided in their superfamilies.

Superfamily	n	%*
(R00–R99): Symptoms, signs, and abnormal clinical and laboratory findings, not elsewhere classified	1237	14.7%
(I00–I99): Diseases of the circulatory system	1123	13.3%
(E00–E90): Endocrine, nutritional, and metabolic diseases	864	10.3%
(M00–M99): Diseases of the musculoskeletal system and connective tissue	692	8.2%
(Z00–Z99): Factors influencing the health status and contact with health services	654	7.8%
(G00–G99): Diseases of the nervous system	522	6.2%
(F00–F99): Mental and behavioral disorders	515	6.1%
(K00–K93): Diseases of the digestive system	511	6.1%
(N00–N99): Diseases of the genitourinary system	348	4.1%
(H00–H59): Diseases of the eye and adnexa	293	3.5%
(L00–L99): Diseases of the skin and subcutaneous tissue	289	3.4%
(D50–D89): Diseases of the blood and blood-forming organs and certain disorders involving the immune mechanism	267	3.2%
(S00–T98): Injury, poisoning, and certain other consequences of external causes	256	3.0%
(U00–U99): Codes for special purposes: COVID-19	197	2.3%
(J00–J99): Diseases of the respiratory system	188	2.2%
(C00–D48): Neoplasms	150	1.8%
Interventions	131	1.6%
(H60–H95): Diseases of the ear and mastoid process	109	1.3%
(A00–B99): Certain infectious and parasitic diseases	61	0.7%
(V01–Y98): External causes of morbidity and mortality	12	0.1%
Total	8419	100.0%

* represents the percentage of the total registered diseases in each group.

All patients, except for 1, used a minimum of one pharmacological treatment with a mean of 8.22 drugs prescribed (SD 3.5), including fixed-dose combinations. The three most prescribed medications were omeprazole, prescribed to 274 patients (56.8%), paracetamol, prescribed to 269 patients (55.8%), and quetiapine, prescribed to 183 patients (37.9%), as seen in Table 4. For a complete list of all the pharmacological prescribed treatments divided into groups according to their ATC, see Supplementary Table S2.

**TABLE 4:** Summary of the 40 most frequent pharmacological treatments.

Drug	n	%
Omeprazole	274	56.8%
Paracetamol	269	55.8%
Quetiapine	183	37.9%
Furosemide	144	29.8%
Acetylsalicylic acid	134	27.8%
Enalapril	109	22.6%
Lorazepam	105	21.7%
Bisoprolol	89	18.4%
Vitamin D and analogs	86	17.8%
Simvastatin	78	16.2%
Sertraline	74	15.3%
Trazodone	69	14.3%
Amlodipine	66	13.7%
Citalopram	62	12.8%
Atorvastatin	61	12.6%
Risperidone	61	12.6%
Metformin	60	12.4%
Ferrous glycine sulfate	60	12.4%
Levothyroxine sodium	60	12.4%
Calcium combinations with vitamin D and/or other drugs	54	11.2%
Mirtazapine	53	10.9%
Memantine	43	8.9%
Losartan	41	8.5%
Metamizole sodium	41	8.5%
Folic acid	40	8.3%
Apixaban	37	7.6%
Fentanyl	35	7.2%
Clopidogrel	34	7.0%
Hydrochlorothiazide	34	7.0%
Insulin glargine	31	6.4%
Acenocoumarol	31	6.4%
Gabapentin	31	6.4%
Pregabalin	29	6.0%
Donepezil	28	5.8%
Rivastigmine	28	5.8%
Latanoprost	27	5.6%
Tramadol	25	5.1%
Levodopa and decarboxylase inhibitor	25	5.1%
Lormetazepam	25	5.1%
Rivaroxaban	21	4.3%

### 3.2 Descriptive analysis of the recommendations and medication-related problems

**FIGURE 2:**
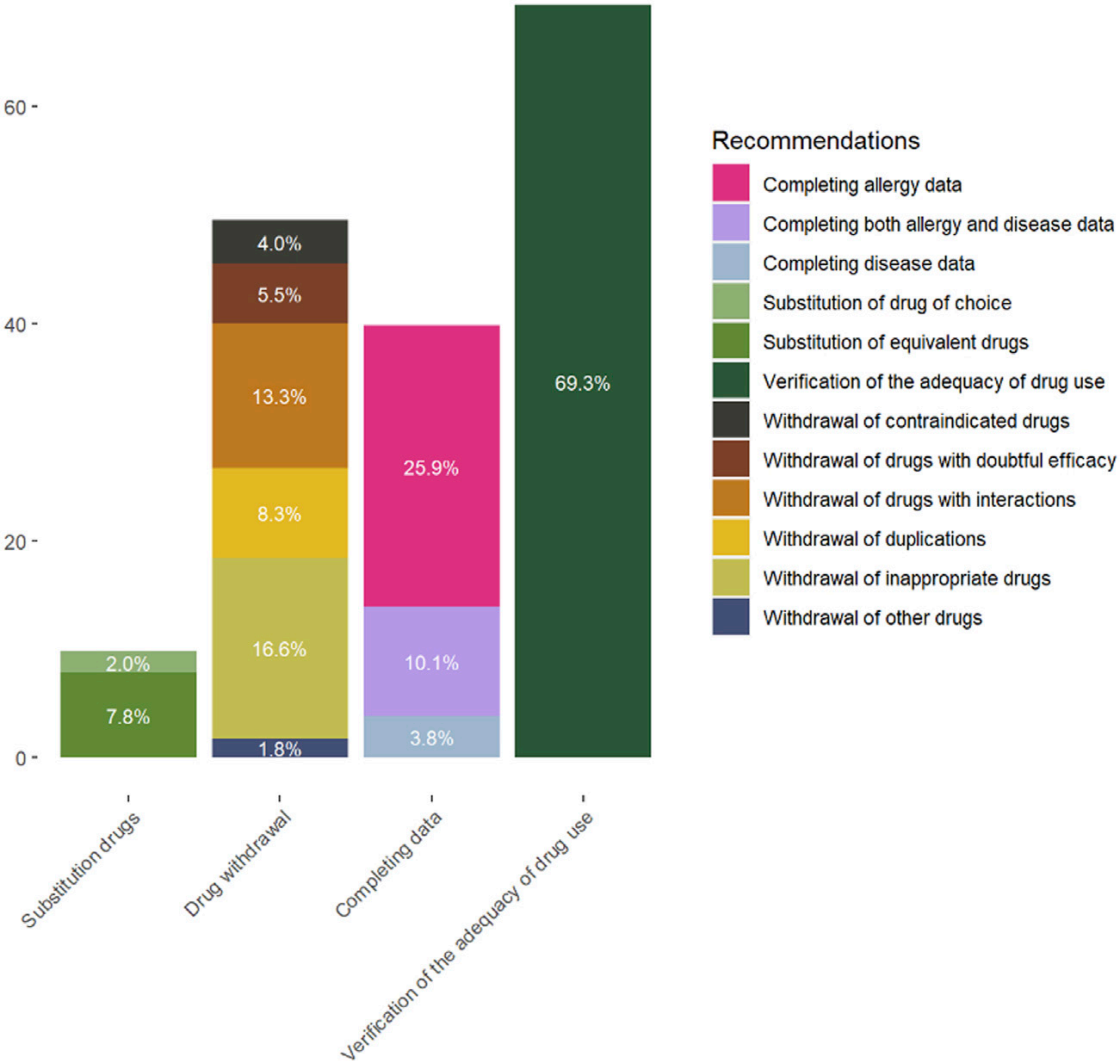
Percentages of the different recommendations subdivided.

A clinical pharmacologist made recommendations for 398 (82.4%) patients. The patients could get various recommendations. In a total of 165 (34.2%) patients, some of the data concerning their HRPs or allergies were absent. The most frequent recommendation was the verification of the adequate use of drugs for 276 (69.3%) patients. The withdrawal of at least one drug was recommended for 197 (49.5%) patients, and substitution of a drug was recommended for 39 (9.8%) patients, as seen in Figure 2.

The MRPs recommended to be withdrawn were due to potential DDIs, therapeutic duplications, contraindications, and drugs deemed inappropriate or of doubtful efficacy. Combining all MRPs, there were 231 (47.8%) in total. Table 4 shows all the MRPs mentioned in the pharmacological review. There was a risk of interactions in 61 (12.6%) patients, with a total of 72 (14.9%) potential DDIs. Of all the potential DDIs, 27 of them included a selective serotonin reuptake inhibitor (SSRI) drug (37.5%), of which tramadol-SSRI was the most common, with 16 (22.2%) potential DDIs in total. Statins and calcium channel blockers were 13 (18.0%) of the potential DDIs, and a combination of different antiarrhythmics and cardiac glycosides was seen in 8 (11.1%) DDIs. Regarding the therapeutic duplications, a prevalence of vitamin D or analogs associated with calcium is seen. Contraindications were seen recurrent in metformin, NSAIDs, and haloperidol. Inappropriate drugs were mostly antipsychotics or benzodiazepines. Lastly, the drugs with doubtful efficacy were often psychostimulant and antivertiginous drugs, as can be seen in Table 5 along with the active ingredients according to their ATC classification.

**TABLE 5:** List of all the medication-related problems mentioned in the pharmacological review.

Potential drug-drug interactions (n, %), (72, 14.9%)		Therapeutic duplications^b^ (n, %), (38, 7.8%)**		Contraindications (n, %), (23, 4.7%)		Inappropriate drugs (n, %), (76, 15.7%)		Drug of doubtful efficacy (n, %), (22, 4.5%)	
Tramadol-SSRI	16 (22.2%)	Vitamin D and analogues	10 (13.1%)	Metformin	5 (21.7%)	Alprazolam	10 (13.1%)	Citicoline	6 (27.2%)
Tramadol-sertraline	8 (11.1%)	Calcium combined with vitamin D or other drugs	8 (10.5%)	Haloperidol	2 (8.7%)	Paroxetine	7 (9.2%)	Betahistine	5 (22.9%)
Tramadol-citalopram	5 (6.9%)	Levothyroxine sodium	4 (5.2%)	Citalopram	2 (8.7%)	Clonazepam	6 (7.9%)	Clebopride	2 (9.1%)
Tramadol-paroxetine	3 (4.1%)	Paracetamol	4 (5.2%)	Dabigatran etexilate	1 (4.3%)	Domperidone	5 (6.5%)	Glutamic acid hydrochloride	1 (4.5%)
Statins-calcium channel blockers	13 (18.0%)	Pregabalin	4 (5.2%)	Amiodarone	1 (4.3%)	Diazepam	5 (6.5%)	Cilostazol	1 (4.5%)
Simvastatin-amlodipine	9 (12.5%)	Quetiapine	4 (5.2%)	Hydralazine	1 (4.3%)	Digoxin	4 (5.2%)	Trimetazidine	1 (4.5%)
Simvastatin-diltiazem	3 (4.1%)	Trazodone	4 (5.2%)	Hydrochlorothiazide	1 (4.3%)	Doxazosin	4 (5.2%)	Naftidrofuryl	1 (4.5%)
Diltiazem-atorvastatin	1 (1.3%)	Omeprazole	3 (3.9%)	Spironolactone	1 (4.3%)	Metoclopramide	3 (3.9%)	Diosmin	1 (4.5%)
Acenocumarol	11 (15.3%)	Folic acid	3 (3.9%)	Enalapril	1 (4.3%)	Solifenacin	3 (3.9%)	Megestrol	1 (4.5%)
Acenocumarol-statins	6 (8.3%)	Furosemide	2 (2.6%)	Atorvastatin	1 (4.3%)	Potassium clorazepate	3 (3.9%)	Mirabegron	1 (4.5%)
Acenocumarol-levotyroxin	5 (6.9%)	Diltiazem	2 (2.6%)	Raloxifene	1 (4.3%)	Pentoxifylline	2 (2.6%)	Prunus africanae cortex	1 (4.5%)
SSRI and other drugs	11 (15.3%)	Bisoprolol	2 (2.6%)	Mirabegron	1 (4.3%)	Bisoprolol	2 (2.6%)	Levosulpiride	1 (4.5%)
Donezepil-citalopram	4 (5.5%)	Losartan	2 (2.6%)	Diclofenac	1 (4.3%)	Fesoterodine	2 (2.6%)		
Citalopram-amytriptiline	1 (1.3%)	Clobetasol	2 (2.6%)	Aceclofenac	1 (4.3%)	Hydroxyzine	2 (2.6%)		
Citalopram-domperidone	1 (1.3%)	Tramadol and paracetamol	2 (2.6%)	Dexketoprofen	1 (4.3%)	Clomethiazole	2 (2.6%)		
Citalopram-haloperidol	1 (1.3%)	Oxcarbazepine	2 (2.6%)	Alendronic acid	1 (4.3%)	Ursodeoxycholic acid	1 (1.3%)		
Citalopram-hydralazine	1 (1.3%)	Gabapentin	2 (2.6%)	Galantamine	1 (4.3%)	Liquid paraffin	1 (1.3%)		
Citalopram-sulpiride	1 (1.3%)	Levodopa and decarboxylase inhibitor	2 (2.6%)			Metformin	1 (1.3%)		
Citalopram-tapentadol	1 (1.3%)	Mirtazapine	2 (2.6%)			Hydralazine	1 (1.3%)		
Donezepil-escitalopram	1 (1.3%)	Pantoprazole	1 (1.3%)			Telmisartan and diuretics	1 (1.3%)		
Antiarrythmics and cardiac glicosides	8 (11.1%)	Vitamin B and acid folic	1 (1.3%)			Simvastatin	1 (1.3%)		
Amiodarone-beta blockers	2 (2.7%)	Hydrochlorothiazide	1 (1.3%)			Atorvastatin	1 (1.3%)		
Bisoprolol-alfuzosine	1 (1.3%)	Torasemide	1 (1.3%)			Febuxostat	1 (1.3%)		
Diltiazem-amlodipine	1 (1.3%)	Timolol and thiazides	1 (1.3%)			Trihexyphenidyl	1 (1.3%)		
Diltiazem-bisoprolol	1 (1.3%)	Captopril	1 (1.3%)			Haloperidol	1 (1.3%)		
Diltiazem-digoxin	1 (1.3%)	Enalapril	1 (1.3%)			Benzodiazepine	1 (1.3%)		
Flecainide-bisoprolol	1 (1.3%)	Fluticasone	1 (1.3%)			Bromazepam	1 (1.3%)		
Verapamil-propanolol	1 (1.3%)	Budesonide	1 (1.3%)			Loprazolam	1 (1.3%)		
Enalapril	5 (6.9%)	Timolol	1 (1.3%)			Zolpidem	1 (1.3%)		
Enalapril-potassium	3 (4.1%)	Latanoprost	1 (1.3%)			Amitriptyline	1 (1.3%)		
Enalapril-eplerenone	1 (1.3%)	Bimatoprost	1 (1.3%)			Trazodone	1 (1.3%)		
Enalapril-lithium	1 (1.3%)								
Other drugs^a^	8 (11.1%)								

*n* = total number of drugs with a related problem for each category in the pharmacological review.

^a^ Other 8 DDIs: Simvastatin–carbamazepine (2)/amiodarone (1)/gemfibrozil (1), NSAIDs–acetylsalicylic acid (1), lamotrigine–valproic acid (1), omeprazole–cilostazol (1), and clozapine–carbamazepine (1).

^b^ The therapeutic duplications are listed double since both drugs were noted. The drugs could be the same or from the same therapeutic family.

## 4 Discussion

The main objective of this study was to describe institutionalized patients and systematically review their medication plans in nursing homes in Catalonia. The results showed a high prevalence of HRP in all patients, with a mean of 8.22 prescribed drugs per patient. This is similar to other studies in Europe (Pasina et al., 2020; Reinhild Haerig et al., 2023). More than 80% of the patients received recommendations, and for 50%, at least one drug was recommended to be withdrawn due to MRPs. These results confirm the challenge of the most fragile patients in nursing homes, with a high number of prescribed medications, raising the possibility of MRPs, PIMs, risk of ADRs, and lack of interventions to improve adequate polypharmacy. This intervention gave specific recommendations to each patient to reduce MRPs, PIMs, ADRs, and polypharmacy. This should help resolve potential MRPs and prevent medication errors.

### 4.1 Descriptive analysis of institutionalized patients in nursing homes

The majority of patients were female individuals (72.0%) with a mean age of 86.3 years, which is similar to other comparable European studies (San-José et al., 2014; Schneider et al., 2019; Burato et al., 2021; Troncoso-Mariño et al., 2021). This was expected since female people have a longer life expectancy (Eurostat, 2023). In a nursing home in Italy, the prevalence of female individuals was likewise elevated, being 78.3% and 74.9% of patients with and without dementia, respectively (Pasina et al., 2020).

The number of HRPs was also very high, with a mean of 17.4 diseases, which agrees with the AMG values and the type of patient that is mostly admitted to nursing homes. It also highlights the risks of the frailer elderly and their association with polypharmacy and increased MRPs. This does not correlate with the low percentage of complex chronic patients or model of attention to advanced chronicity described in this study. The cause of this under-registration may be due to the complexity and time needed to go through different scales and classify a patient as complex chronic or of advanced chronicity.

According to the HRPs, the proportion of dementia among the residents living in nursing homes is high. Alzheimer’s or dementia was observed in 52.8% of the patients, and patients with symptoms or signs involving cognitive functions and awareness were 30.2%. These diseases are important to take into account when reviewing the medication since they are more likely to be prescribed antipsychotic drugs, leading to a higher risk of MRPs (Taxis et al., 2017; Pasina et al., 2020).

There is an excessive number of prescribed drugs in institutionalized patients in Catalonia, with a mean of 8.22 drugs, similar to nursing homes in Italy, where some regions show polypharmacy in 80.3% of the inpatients in nursing homes (Pasina et al., 2020), or Switzerland, with polypharmacy in 85.5% and a mean number of drugs of 9.4 (Schneider et al., 2019). The excessive number of prescribed drugs is consistent with other parts of the world, such as in Australia, where more than 50% of nursing home residents use nine or more regular medications, leading to the proposal of a simplified medication regimen to reduce the medication burden (Bell et al., 2021).

The three most prescribed drugs were proton pump inhibitors (PPIs), analgesics, and antipsychotics or tranquilizers. This pattern is similar to the not institutionalized Spanish population (Troncoso-Mariño et al., 2021) but with a superior number of prescribed drugs (Cebrino and Portero de la Cruz, 2023). The sequence of most prescribed drugs is similar to that in other European countries, with the most frequent drugs being analgesics (paracetamol and metamizole), diuretics (torasemide), PPIs (pantoprazole), and tranquilizers (quetiapine) (Schneider et al., 2019). PPI use is only considered appropriate for current gastric or duodenal disorders or the prevention of NSAID effects (Zito et al., 2023). Therefore, most of the patients in our study do not meet the criteria for PPI use. Psychotropic use is higher in our study group than in nursing home reports from other countries, such as Australia (69.9%) and Germany (71.1%) (Taxis et al., 2017), but it is similar to that in Italy (Pasina et al., 2020). In nursing homes in Norway, after comparing the prescription of a psychotropic drug at baseline and after 6 months, there was a significant difference with an increase in prescribed antidepressants, atypical antipsychotics, anxiolytics, and sedatives/hypnotics (Callegari et al., 2021).

### 4.2 Descriptive analysis of the given recommendations and medication-related problems in nursing homes

A patient’s clinical state changes over time, and it is necessary to review their treatment systematically. With a multidisciplinary team in nursing homes with both clinical pharmacologists and geriatricians, it is possible to carry out a comprehensive geriatric assessment, including a thorough review of the medication. The reason is that patients in nursing homes are mostly in a situation of advanced fragility and are candidates for deprescription to avoid ADRs and MRPs. With the multidisciplinary approach, recommendations were given, and MRPs were identified. The clinical decision support system in Catalonia helps improve these changes, but since only 28.0% of the alerts were accepted, discussion is needed on improving the approval rate of these warnings (Pons-Mesquida et al., 2021). PREFASEG and Self-Audit are tools used in Catalonia to detect MRPs like potential DDIs, but there are other tools, such as DDI-Predictor or Medscape, that are used by different health professionals in diverse situations (Marcath et al., 2018; Moreau et al., 2021). Prescription errors are more frequent in frail older populations, and systems to detect prescription errors are needed. Interventions to optimize prescription are time-consuming and not always included in routine clinical care. Some consider that appropriately trained clinical pharmacists and communication-technology support are required (Lavan et al., 2016). A recent article also considers that the engagement of clinical pharmacists can prevent MRPs, collaborating with a multidisciplinary team and other international organizations, thereby achieving patient-centered healthcare in Europe and a positive impact (Urbańczyk et al., 2023). Transition of care with appropriate medication reconciliation could lead to fewer MRPs. Medication reconciliation is predominantly made by physicians and nurses, but it could also be provided by clinical pharmacists in some countries (Stuhec and Batinic, 2023). This underlines the importance of a multidisciplinary approach taking into account that, in Spain, clinical pharmacology is a medical specialty that can also prescribe and make medication changes.

The MRPs in this pharmacological review of drugs that were recommended to withdraw was 47.8%. The majority of potential DDIs included SSRIs, tramadol, statins, acenocoumarol, and calcium channel blockers. Some of these potential interactions have also been described by other authors, such as SSRIs (Pasina et al., 2020), statins (Lion et al., 2023), and warfarin (Neidecker et al., 2012). This is a concern since tramadol increases the potential of seizures when it is administered with SSRIs, serotonin/norepinephrine reuptake inhibitors (SNRIs), and tricyclic antidepressants, among others. They may also cause a life-threatening serotonin syndrome with these interactions (Spanish Agency for Medicines and Health Products, 2021). When statins and calcium channel blockers are administered in combination, the most important thing is to control or not exceed the recommended doses due to the increased risk of myopathy and rhabdomyolysis (Piccoliori et al., 2021). Levothyroxine and statins are drugs included in medications that can potentiate the anticoagulant effect of acenocoumarol, and the combination of different antiarrhythmics is not recommended in older patients due to the greater arrhythmogenic risk (Verhovsek et al., 2008; Neidecker et al., 2012; Iniesta-Navalón et al., 2019). This is without taking into consideration the risk of hypotension, sedations, and, consequently, falls (Piccoliori et al., 2021).

A European study reported higher MRP rates, with the most frequent potentially severe DDIs being psychotropic drugs with additive effects on QTc prolongation, associations of angiotensin-converting enzyme inhibitors or angiotensin II receptor blockers with potassium supplements, increasing the risk of hyperkalemia, and SSRI/SNRI with antiplatelets, increasing the risk of hemorrhage (Pasina et al., 2020). A study performed in a region in Italy showed that the three most frequent DDIs were antidepressants–anxiolytics (11.9%), SSRIs–aspirin (7.4%), and antidiabetics–β-adrenoceptor blockers (5.3%) (Burato et al., 2021).

Regarding the therapeutic duplications, excluding the prevalence of vitamin D or analogs associated with calcium, the rest was observed to be due to patients who are undergoing drug dose adjustments or changes. Both PREFASEG and Self-Audit detect therapeutic duplication, which helps explain the low percentage of duplications detected in this medication review (Pons-Mesquida et al., 2021; 2022). In a recent study done in a pediatric health system, where they designed clinical decision support to reduce therapeutic duplication with acetaminophen and ibuprofen, they saw a therapeutic duplication reduction, but it was associated with high rates of user frustration and alert fatigue (E Dawson et al., 2023).

There were drugs that were contraindicated, such as metformin and NSAIDs, due to chronic renal failure. During this intervention, the renal function was reviewed, and possible contraindications or dose adjustments were recommended according to glomerular filtration. If there was no determination during the last year, the convenience of performing an analysis was indicated (Wood et al., 2018; Writing Group for the CKD Prognosis Consortium et al., 2023). Another cross-sectional study on medication burden and inappropriate prescription risk among the elderly with advanced chronic kidney disease showed that at least one contraindicated drug was prescribed to 10.8% of all patients, and the most frequently prescribed were rilmenidine (16.5%), rosuvastatin (6.5%), alfuzosin (5.8%), and buflomedil (3.6%) (Roux-Marson et al., 2020). Antidepressants, antipsychotics, and benzodiazepines were mainly due to their anticholinergic effect and the increased risk of falls. This is similar to drugs deemed inappropriate or of doubtful efficacy; adding more prescribed drugs with anticholinergic effects increases the possibility of orthostatic hypotension and increased risk of falls (Catalan Health Service. Department of Health., 2020). This illustrates the main reasons why in frail patients, one must be even more consistent with the prior risk–benefit balance.

## 5 Strengths and limitations

There were multiple strengths in this study. With the intervention, this study provided specific recommendations to each patient to reduce MRPs, PIMs, ADRs, and polypharmacy. The medical review was done by a medical doctor specializing in clinical pharmacology, who could change the prescriptions when needed, make an accurate medication review, and give individual recommendations. The availability of a common informatic system helped review the prescription registry and made it possible to act in a coordinated way between nursing homes and primary and hospital care. It was considered an advantage working on this project with primary care professionals, nursing homes, and medical doctors in geriatrics and clinical pharmacology, creating a multidisciplinary team with an agreed final decision.

However, there were also multiple limitations to the study. The intervention was conducted in one urban area, so the findings should be extrapolated to other regions or countries with caution. We gathered data from five different nursing homes, covering 22.3% of the population in the northern area of Barcelona, in Catalonia, so this may be representative of areas with a similar socioeconomic level. Second, the high changes in residents and the variability in the different nursing homes can make the interpretation and extrapolation of the data difficult (Ordovás et al., 2020; Rada, 2020). Third, since the intervention was carried out in routine clinical practice, some information is lacking, such as all non-pharmacological treatments, treatments not registered, or treatments not financed by the public health system, nor is there information on drug adherence. Additionally, the intervention was performed during the COVID-19 pandemic. The pandemic disrupted healthcare systems, leading to delays that influenced daily practice conditions and resulted in serious outcomes for elderly patients. This may have impacted our findings, given that the altered healthcare system complicated the clinical management of elderly populations. For instance, there was no adequate optimization of psychotropic drugs, in line with the social isolation and loneliness experienced in the pandemic, which led to depression, anxiety, cognitive decline, and exacerbation of pre-existing health conditions (Ministry of Health, Spain, 2020). To confirm these results and provide a broader international picture, similar assessment and prospective studies with a control group and out-of-the-pandemic context should be repeated in elderly people in different regions.

## 6 Conclusion

A high prevalence of health-related problems and number of prescribed drugs were observed through medication review in nursing homes. Many recommendations were made, confirming the increasing incidence of polypharmacy and the need for standardized interventions to reduce medication-related problems and the number of prescribed drugs. Specific interventions targeting nursing homes could lower the percentages of medication-related problems. Tools and clinical decision support systems help in reviewing the medication of the patients. This should be addressed with a multidisciplinary team approach, including general practitioners, geriatric assessment, a clinical pharmacist, and a clinical pharmacologist.

## Data Availability

The original contributions presented in the study are included in the article/Supplementary Material; further inquiries can be directed to the corresponding author.
